# Persistent Inflammation and Endothelial Activation in HIV-1 Infected Patients after 12 Years of Antiretroviral Therapy

**DOI:** 10.1371/journal.pone.0065182

**Published:** 2013-06-03

**Authors:** Frederikke F. Rönsholt, Henrik Ullum, Terese L. Katzenstein, Jan Gerstoft, Sisse R. Ostrowski

**Affiliations:** 1 Department of Infectious Diseases, Rigshospitalet, Copenhagen, Denmark; 2 Department of Clinical Immunology, Rigshospitalet, Copenhagen, Denmark; New York University, United States of America

## Abstract

**Objective:**

The study investigated markers of inflammation and endothelial activation in HIV infected patients after 12 years of successful combination antiretroviral treatment (cART).

**Methods:**

Inflammation and endothelial activation were assessed by measuring levels of immunoglobulins, β2-microglobulin, interleukin (IL) 8, tumor necrosis factor α (TNFα), vascular cell adhesion molecule-1 (sVCAM-1), intercellular adhesion molecule-1 (sICAM-1), sE-Selectin, and sP-Selectin.

**Results:**

HIV infected patients had higher levels of β2-microglobulin, IL-8, TNFα, and sICAM-1 than uninfected controls, and HIV infected patients lacked correlation between platelet counts and sP-Selectin levels found in uninfected controls.

**Conclusion:**

Discrete signs of systemic and vascular inflammation persist even after very long term cART.

## Introduction

Systemic inflammation and immune activation are hallmarks of human immunodeficiency virus (HIV) infection and even after long term combination antiretroviral treatment (cART) some degree of low-grade inflammation persists. As part of the inflammatory response to HIV infection endothelial activation and release of vascular adhesion molecules is seen and several different markers reflecting ongoing inflammation and endothelial activation are increased in HIV infected patients even after long term cART. This low-grade inflammation has been suggested to contribute to the increased incidence of cardiovascular and thromboembolic events in treated HIV infected patients [1] as some inflammatory markers i.e. CRP, fibrinogen, D-dimer, IL-6, sICAM-1, and sE-Selectin have been shown to predict cardiovascular events in HIV infected and uninfected individuals [2], [3].

The present study measured markers of residual inflammation, platelet activation and vascular endothelial activation previously described to be affected by HIV infection and/or predictive for cardiovascular events, and investigated their correlation to viraemia, current CD4 count, and cardiovascular risk factors in a cohort of HIV infected patients who have received cART continuously since 1996–97 and responded to treatment with undetectable viral loads.

## Materials and Methods

### Study Population

The study was conducted at the Department of Infectious Diseases and the Department of Clinical Immunology at Rigshospitalet (Copenhagen, Denmark). The study population comprises HIV infected patients included in the period September 1997–August 1998 on the basis of having reproducible plasma HIV RNA levels <200 copies/mL after starting cART. One-hundred-and-one patients entered the study in 1997–98– at follow up in 2009, 17 of those had died and 13 were lost to follow up, leaving 71 patients. One of these patients experienced a viraemic bleep of 8888 copies/mL on the day of sampling and was excluded, leaving 70 patients who participated in the present study. The patients who died during the follow up period and their causes of death have been described previously [4].

Blood samples were obtained in conjunction with the patients’ routine visits to the out-patient clinic and background data were obtained from the patients’ charts and the Danish HIV Cohort. All patients gave written informed consent and the study was approved by The Comities on Biomedical Research Ethics for the Capital Region in Denmark (journal number H-C-2008-077).

As treatment interruptions have never been part of the Danish treatment guidelines, the patients received cART continually since inclusion, although the drug combinations have changed over the years due to introduction of new drug combinations, side effects etc.

The control group consisted of 16 age- and gender matched healthy volunteers from the Danish Blood Donor Corps known to be HIV, hepatitis A and B seronegative.

### Hematological Parameters, Immunoglobulins and β2-microglobulin

Hemoglobin, platelets, lymphocytes, IgA, IgG, IgM and β2-microglobulin were measured by standardized methods at the hospital’s central laboratory.

### Ultra Sensitive HIV RNA Measurements

Quantification of HIV RNA was performed at the AIDS laboratory, Rigshospitalet, on EDTA plasma by an ultrasensitive method based on a modified Amplicor assay (Cobas Amplicor HIV-1 monitor test, version 1.5 ultrasensitive assay, Roche Diagnostics, Branchburg, New Jersey, USA) to reach a lower level of detection of 2.5 copies/mL as used and described in detail in other studies [5]. HIV RNA measurements of <2.5 copies/mL were recorded as 2.4 copies/mL.

### Soluble Markers of Inflammation and Vascular Activation

All markers were measured in thawed EDTA plasma using commercially available kits according to the manufacturer’s instructions.

IL-8 and TNFα were measured by quantitative sandwich enzyme immunoassay technique (Quantikine Immunoassay, R&D Systems, Inc., Minneapolis, USA). Samples from HIV infected patients and controls were measured in duplicates and uniplicates respectively. Undetectable values of IL-8 were recorded as the specified minimal detectable level of 3.5 pg/mL. Intra-assay variance on optical densities was 11% and 9% for IL-8 and TNFα respectively.

Soluble ICAM-1 (CD54), VCAM-1 (CD106), E-Selectin (CD62E), and P-Selectin (CD62P) were measured by a bead-based multiplex kit (Fluorokine MAP, R&D Systems, Inc., Minneapolis, USA) on a Luminex-100 analyzer (Luminex Corporation, Texas, USA).

### Statistics

Statistical analyses were conducted using SPSS 11.5 and GraphPad Prism 5.03.

Medians were compared using Mann Whitney’s test. Correlation analyses were performed using Spearman’s rank correlation. For comparing the number of patients above the 75th percentile of a given parametre against the number of patients below Chi squared test was used.

P values <0.05 were considered significant.

## Results

### Patients

Of the 70 HIV infected patients who participated in the study 64 were male and 68 were Caucasians. The median age was 55 years. The median baseline CD4 count was 0.19×10^9^/L and the median CD4 count at follow up was (current) 0.63×10^9^/L. The patients had been diagnosed with HIV for a median of 230 months (range 143–328) and had received cART for a median of 150 months (range 143–175). Nineteen patients were diagnosed with AIDS defining events and five had chronic hepatitis C infection.

At the date of sampling, 45 patients had HIV RNA <2.5 copies/mL, 17 patients had HIV RNA between 2.5 and 20 copies/mL, and 8 patients had HIV RNA between 20 and 128 copies/mL.

Plasma HIV RNA was measured regularly during the course of treatment (between 31 and 68 times) and was below 40 copies/mL in a median of 86.7% (IQR 74.7–93.6) of the measurements. Seven patients experienced no viraemic bleeps in the entire study period.

In the control group the median age was 56 years and 14 out of 16 participants were male.

### Hemoglobin, Lymphocytes and Immunoglobulins

HIV infected patients had higher lymphocyte counts (median 2.15×10^9^/L vs. 1.77×10^9^/L, p = 0.028) and lower levels of IgA (2.44 g/L vs. 2.98 g/L, p = 0.026) compared to controls, though both were within normal reference intervals. Hemoglobin, IgG and IgM levels did not differ between HIV infected patients and controls.

### β2-microglobulin, Cytokines and Endothelial Markers

**Figure 1:**
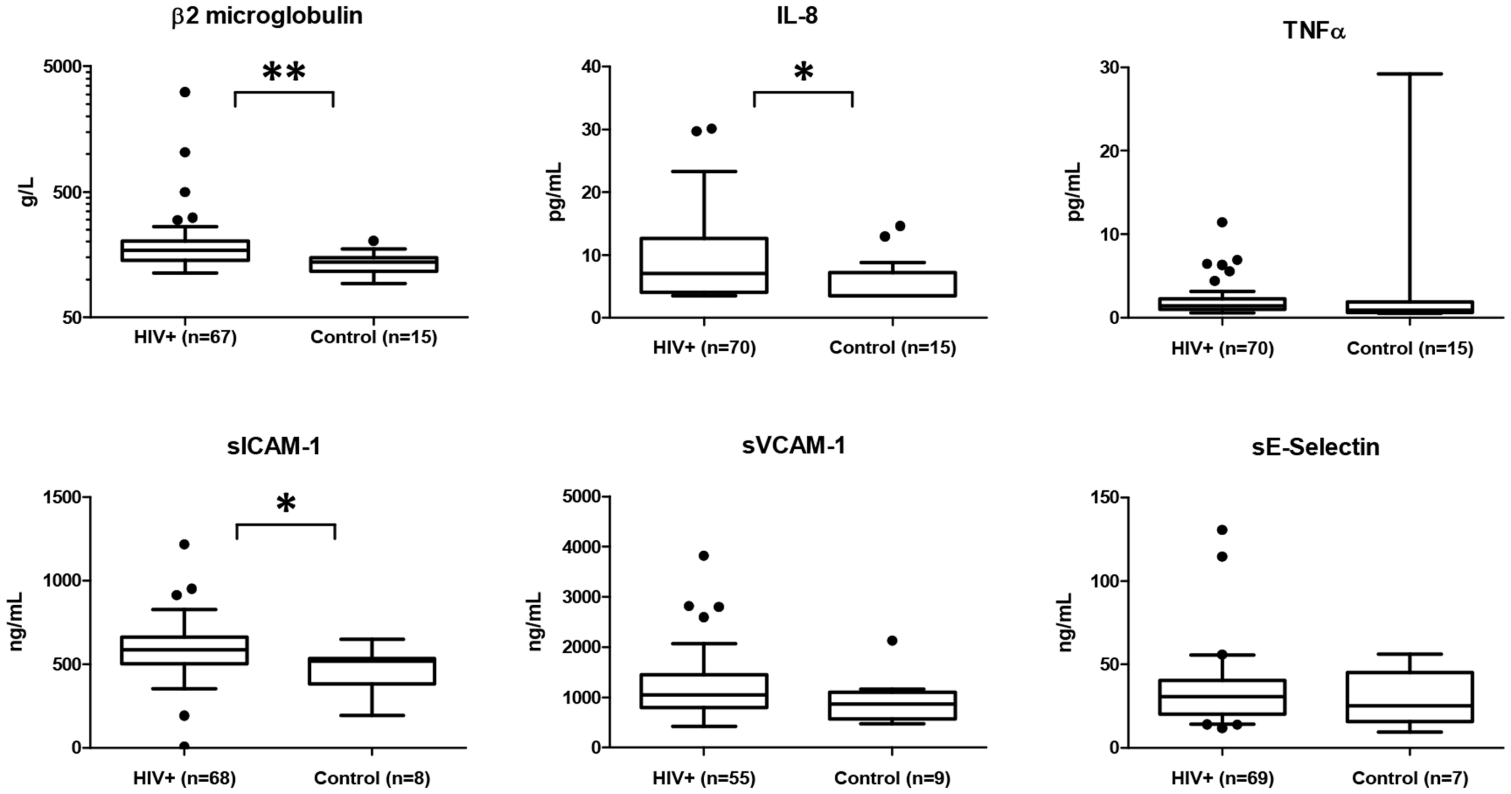
Levels of β2-microglobulin, IL-8, TNFα, sICAM-1, sVCAM-1 and sE-Selectin in HIV infected patients (HIV+) after 12 years of successful cART compared to HIV negative controls. Please note that β2-microglobulin is shown on a logarithmic Y-axis. Means were compared using Mann-Whitney test and depicted as boxplots with Tukey whiskers (1.5 times the interquartile distance or to the highest or lowest point, whichever is shorter). * = p<0.05, ** = p<0.001.

Levels of β2-microglobulin, IL-8, TNFα, sICAM-1, sVCAM-1, and sE-Selectin are shown in Figure 1. HIV infected patients had higher levels of β2-microglobulin, IL-8, and sICAM-1 as compared to controls.

One TNFα measurement among the controls was extraordinarily high (29.19 pg/mL) and may have been an erroneous measurement, as this person did not show any other biochemical signs of excess inflammation.

If this value is excluded from the analysis TNFα levels are significantly higher in HIV infected patients than controls (p = 0.026).

In β2-microglobulin, TNFα, IL-8, and sICAM-1 several patients had values below the highest value of the controls.

The patients with elevated levels of β2-microglobulin, TNFα, IL-8, and sICAM-1 (above versus below the 75^th^ percentile) were generally not the same patients. However, there was a significant overlap between patients with elevated TNFα and patients with elevated β2-microglobulin (Chi squared p = 9.16*10^−5^), and a less pronounced, but statistically significant (Chi squared p = 0.028), overlap between patients with elevated levels of IL-8 and sICAM-1.

### Platelets and sP-Selectin

**Figure 2:**
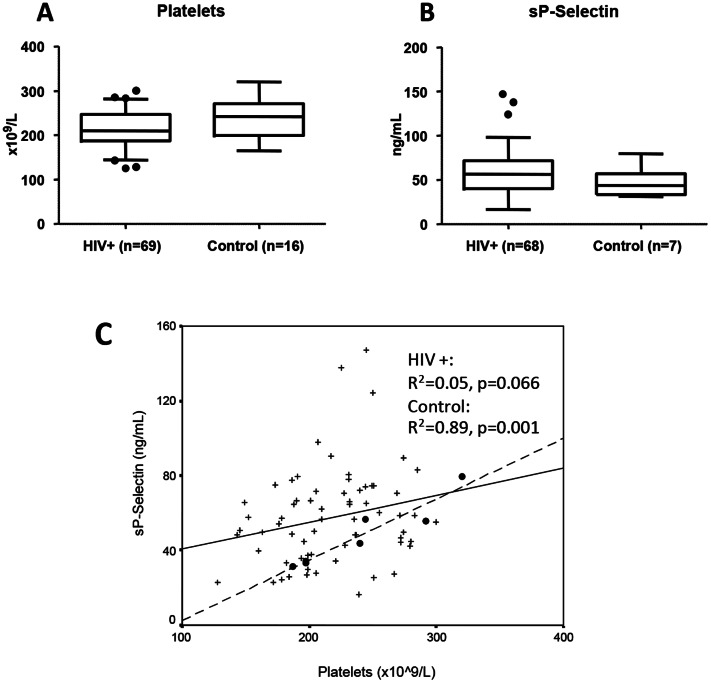
Levels of platelets (A) and sP-Selectin (B) in HIV infected patients (HIV+) after 12 years of successful cART compared to HIV negative controls. Means were compared using Mann-Whitney test and depicted as boxplots with Tukey whiskers (1.5 times the interquartile distance or to the highest or lowest point, whichever is shorter). * = p<0.05, ** = p<0.001. Below (C), scatter plot of correlation between platelet and sP-Selectin concentrations in HIV infected patients (+, full line) and healthy controls (•, dotted line). R^2^ and P values were calculated using linear regression.

Levels of platelets and sP-Selectin and the correlation between platelet and sP-Selectin concentrations in controls and HIV infected patients are shown in Figure 2A and B. Levels of sP-Selectin were similar in HIV infected patients and controls but HIV infected patients tended to have lower platelet counts (p = 0.072) compared to controls. Only in controls did levels of sP-Selectin correlate with platelet count (Figure 2C).

### Association to Residual Viraemia and Current Total CD4 Count

Within the group of HIV infected patients β2-microglobulin, IL-8, TNFα, sICAM-1, sVCAM-1, sE-Selectin, and sP-Selectin did not correlate to current total CD4 count or levels of residual viraemia (not shown).

When separating the patients into groups according to being above or below highest control value there was no correlation between β2-microglobulin, IL-8, and sICAM-1 and viraemia or current CD4 count (not shown).

### Cardiovascular Risk Factors

When comparing the HIV infected patients who had a history of smoking (n = 32) (current or previous tobacco use) with those who had never smoked (n = 39), the smokers had slightly higher levels of sICAM-1 (median 634 ng/mL vs. 560 ng/mL, p = 0.018) but similar levels of β2-microglobulin, IL-8, TNFα, sVCAM-1, sE-Selectin, and sP-Selectin (data not shown). When stratifying the HIV infected patients according to diagnosed hypertension, ongoing statin-treatment or treatment with abacavir containing cART regimes no differences in any of the investigated markers were revealed (data not shown).

When separating the patients into two groups according to β2-microglobulin, TNFα, IL-8, and sICAM-1 being above or below the 75th percentile– and comparing the percentage of patients with hypertension, history of smoking, and statin treatment, no differences were found apart from the previously found correlation between smoking and elevated sICAM-1.

## Discussion

The principal findings of the present study were: i) Even after very long term cART, HIV infected patients had discrete signs of persisting systemic and vascular inflammation compared to healthy controls assessed by levels of β2-microglobulin, IL-8 and sICAM-1, and ii) markers of inflammation were not associated with residual viraemia, current total CD4 count or cardiovascular risk factors except for a moderate association between smoking and higher sICAM-1 levels.

β2-microglobulin levels fall during the first months of cART [6], but the present study showed persistently increased levels of β2-microglobulin in HIV infected patients compared to controls after long term cART.

TNFα levels are elevated in untreated HIV infected patients and are diminishes by cART [7]. In the present study TNFα levels were also elevated after 12 years of cART compared to controls which is consistent with the findings of other studies with shorter follow up that found elevated levels compared to healthy controls after 78 weeks of cART [7].

The present study found higher plasma concentrations of IL-8 in HIV infected patients as compared to controls. A previous study have reported that serum IL-8 levels reduce during 6 months of cART but remain elevated as compared to controls [8]. In contrast, one study showed plasma IL-8 levels to be normalized after only 4 months of cART [9].

Passage of leukocytes through the vascular endothelium is an early inflammatory event and selectins mediate their rolling over the endothelium while adhesion molecules such as VCAM and ICAM mediate adhesion and transmigration [10]. Endothelial activation assessed by increased levels of adhesion molecules have been proposed as a contributing factor to the increased risk of cardiovascular events among treated and untreated HIV patients [11]. In the present study, HIV infected patients had higher levels of sICAM-1, but not significantly higher levels of sVCAM-1, sE-Selectin or sP-Selectin, though the low number of controls may introduce a type II error. Other studies investigating levels of circulating adhesion molecules have reported elevated levels of sVCAM-1 and s-ICAM1 after 9 months of cART [12] and elevated levels of sVCAM-1, but not sICAM-1, sE-Selectin or sP-Selectin after a median of 10 years of cART [13] respectively. Taken together these results suggest that vascular inflammation does persist even after very long term cART.

sP-Selectin is derived from both platelets and endothelial cells [10]. In the present study the concentration of sP-Selectin correlated with platelet counts only in healthy controls suggesting that the contribution to sP-Selectin from the endothelial cells was relatively increased in HIV infected patients due to endothelial activation. Alternatively, platelet dysfunction and exhaustion may hypothetically explain the lack of correlation in the HIV infected patients. Landrø et al. [14] found elevated levels of sP-Selectin in HIV infected patients after 2 years of cART with no correlation to platelet count, and Corrales-Medina et al. [15] found normalized levels of CD62P (P-Selectin) expression on platelets from cART treated HIV infected patients, which further supports the theory that excess amounts of circulating sP-Selectin at least in part origins from activated endothelial cells.

It is important to note that the signs of persistent inflammation in these patients, although present in many branches of the immune system, are very discrete, and some patients have in fact normalized levels of all of the examined inflammatory parameters. None of the examined inflammatory markers correlated to current CD4 counts or viraemia and some were not affected at all: IgG levels were comparable to those of healthy controls in contrast to another study that have shown a decline, but not normalization, of IgG levels after two years of cART [16]. Hemoglobin, although not an inflammatory marker per se, can be an indicator of immune activation and inflammation, and in the present study hemoglobin levels were similar between HIV infected patients and controls.

The strengths of the present study are the long follow up period of uninterrupted treatment and the relatively large number of patients in a heterogeneous population. The weaknesses are the cross sectional design and the small number of controls due to resource limitations. Larger and longitudinal studies are needed to firmly establish the degree and clinical relevance of residual inflammation in cART treated HIV infected patients.

In conclusion, this study demonstrated discrete signs of ongoing systemic and vascular inflammation affecting several branches of the immune system in HIV infected patients after 12 years of successful cART.
